# Invasive ductal breast cancer molecular subtype prediction by MRI radiomic and clinical features based on machine learning

**DOI:** 10.3389/fonc.2022.964605

**Published:** 2022-09-12

**Authors:** Weiyong Sheng, Shouli Xia, Yaru Wang, Lizhao Yan, Songqing Ke, Evelyn Mellisa, Fen Gong, Yun Zheng, Tiansheng Tang

**Affiliations:** ^1^ Department of Cardiothoracic Surgery, The First Affiliated Hospital of Wannan Medical College, Wuhu, China; ^2^ First Clinical Medical College, Guangzhou University of Chinese Medicine, Guangzhou, China; ^3^ Department of Hand Surgery, Union Hospital, Tongji Medical College, Huazhong University of Science and Technology, Wuhan, China; ^4^ Department of Science and Technology Research Management, Wuhan Blood Center, Wuhan, China; ^5^ Department of Emergency Surgery, Union Hospital, Tongji Medical College, Huazhong University of Science and Technology, Wuhan, China; ^6^ Department of Radiology, The First Affiliated Hospital of Guangzhou University of Chinese Medicine, Guangzhou, China

**Keywords:** molecular subtypes, MRI, radiomics, breast cancer, three-dimension, machine learning

## Abstract

**Background:**

Most studies of molecular subtype prediction in breast cancer were mainly based on two-dimensional MRI images, the predictive value of three-dimensional volumetric features from dynamic contrast-enhanced magnetic resonance imaging (DCE-MRI) for predicting breast cancer molecular subtypes has not been thoroughly investigated. This study aimed to look into the role of features derived from DCE-MRI and how they could be combined with clinical data to predict invasive ductal breast cancer molecular subtypes.

**Methods:**

From January 2019 to December 2021, 190 Chinese women with invasive ductal breast cancer were studied (32 triple-negative, 59 HER2-enriched, and 99 luminal lesions) in this institutional review board-approved retrospective cohort study. The image processing software extracted 1130 quantitative radiomic features from the segmented lesion area, including shape-based, first-order statistical, texture, and wavelet features. Three binary classifications of the subtypes were performed: triple-negative *vs*. non-triple-negative, HER2-overexpressed *vs*. non-HER2-overexpressed, and luminal (A + B) *vs*. non-luminal. For the classification, five machine learning methods (random forest, logistic regression, support vector machine, naïve Bayes, and eXtreme Gradient Boosting) were employed. The classifiers were chosen using the least absolute shrinkage and selection operator method. The area evaluated classification performance under the receiver operating characteristic curve, sensitivity, specificity, accuracy, F1-Score, false positive rate, precision, and geometric mean.

**Results:**

EXtreme Gradient Boosting model showed the best performance in luminal and non-luminal groups, with AUC, sensitivity, specificity, accuracy, F1-Score, false positive rate, precision, and geometric mean of 0.8282, 0.7524, 0.6542, 0.6964, 0.6086, 0.3458, 0.8524 and 0.7016, respectively. Meanwhile, the random forest model showed the best performance in HER2-overexpressed and non-HER2-overexpressed groups, with AUC, sensitivity, specificity, accuracy, F1-Score, false positive rate, precision, and geometric mean of 0.8054, 0.2941, 0.9744, 0.7679, 0.4348, 0.0256, 0.8333 and 0.5353, respectively. Furthermore, eXtreme Gradient Boosting model showed the best performance in the triple-negative and non-triple-negative groups, with AUC, sensitivity, specificity, accuracy, F1-Score, false positive rate, precision, and geometric mean of 0.9031, 0.9362, 0.4444, 0.8571, 0.9167, 0.5556, 0.8980 and 0.6450.

**Conclusion:**

Clinical data and three-dimension imaging features from DCE-MRI were identified as potential biomarkers for distinguishing between three molecular subtypes of invasive ductal carcinomas breast cancer. In the future, more extensive studies will be required to evaluate the findings.

## Introduction

Breast cancer accounts for about 30% of female cancers worldwide, with a mortality-to-incidence ratio of 15% (1). As the world’s largest developing country, China ranks first in terms of female breast cancer incidence and deaths, accounting for 17.6% and 15.6% of global female breast cancer incidence and deaths, respectively (2). Breast cancer subtyping has important therapeutic implications for the disease’s clinical management. The luminal (A or B), human epidermal growth factor receptor 2 (HER2)-overexpressed, and triple-negative subtypes of breast cancer are the most common molecular subtypes (3). Most invasive breast cancers (70%) are luminal tumors, which respond well to endocrine therapy. HER2-positive tumors are more likely to respond to targeted antibody therapy (4). Although triple-negative cancers are more aggressive and challenging to treat, some respond well to chemotherapy (4, 5). In routine clinical practice, breast cancer subtypes can be identified using genetic array testing or immunohistochemistry markers. Immunohistochemistry necessitates tissue samples, which are usually obtained through a needle biopsy. The subtyping assessment performed on a needle biopsy sample may not represent the tumor entirely due to the small tissue sample size and tumor heterogeneity.

The use of radiological images to characterize breast cancer subtypes has recently progressed. For example, the molecular subtypes of breast cancer are linked to certain qualitative and visual information of imaging characteristics assessed on breast magnetic resonance imaging (MRI), mammography, or ultrasound (6, 7). The usage of MRI to obtain multiparametric data from morphologic and functional signals is becoming more prevalent (8). Several radiomic studies have been conducted in breast cancer research. They are primarily based on DCE-MRI or combined MRI with other imaging modalities, such as PET (9). MRI is the most sensitive imaging modality for detecting, characterization, and accurate extent definition of soft tissue tumors (10, 11). DCE-MRI is particularly useful in determining breast cancer’s anatomic and functional properties (12). Previous radiomic studies of breast cancer have been conducted for invasiveness assessment (13, 14), treatment response (15–17) and recurrence prediction (18, 19), and genomic correlation (18). However, these studies (20–23) were primarily based on texture analysis of two-dimensional images; the predictive value of three-dimensional volumetric features from DCE-MRI for predicting breast cancer molecular subtypes has not been thoroughly investigated. This study aimed to see if features extracted from DCE-MRI three-dimensional imaging analyses and clinical data could be used to predict invasive ductal breast cancer molecular subtypes using machine learning. We are the first to classify three distinct molecular subtypes of invasive ductal breast cancer using three-dimensional volumetric imaging features based on a larger sample from Chinese women.

## Materials and methods

### Patients and clinical information

**Figure 1:**
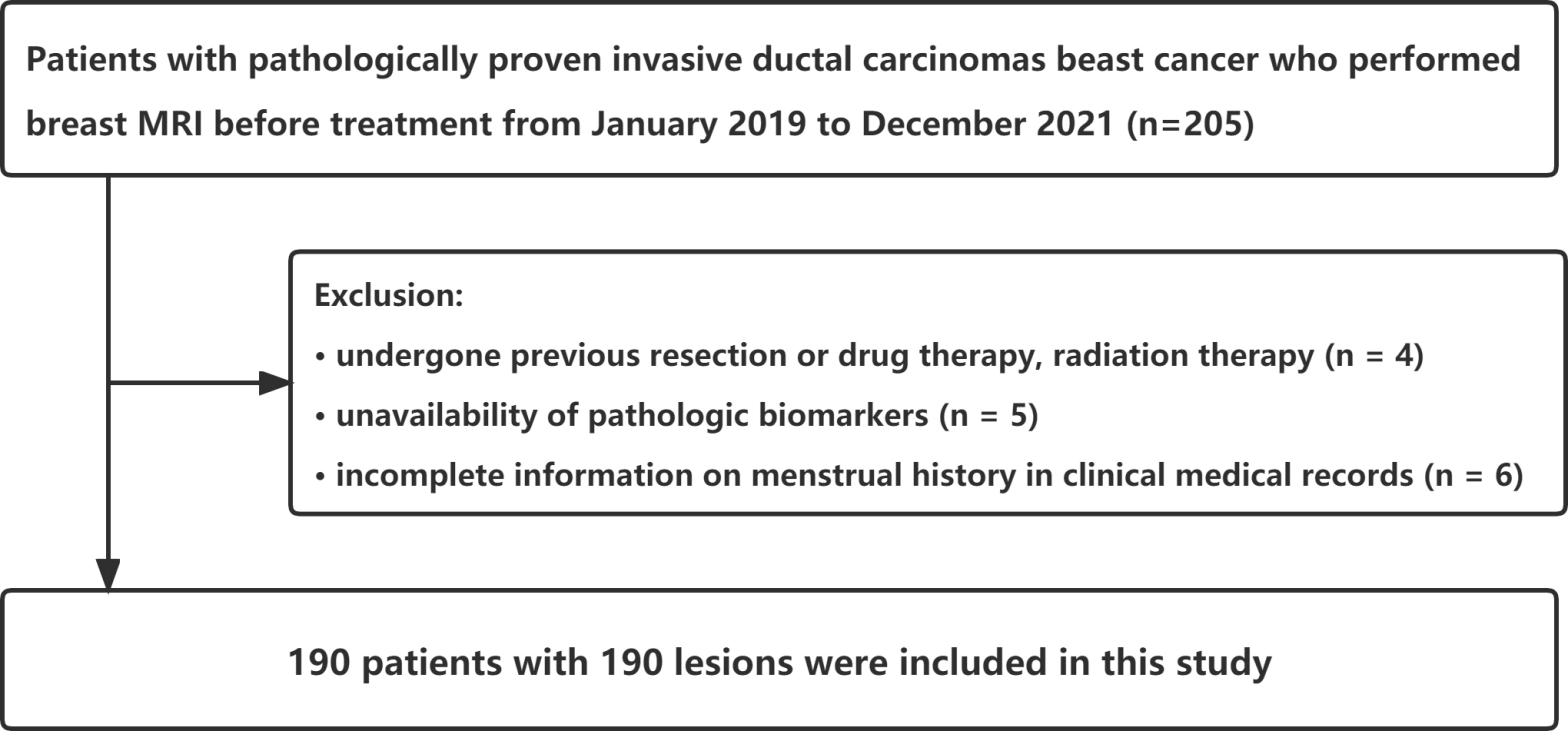
Flowchart of study population with exclusion criteria.

The Ethics Committee of the First Affiliated Hospital of Guangzhou University of Chinese Medicine reviewed and approved this retrospective cohort study (ethics approval number: JY2021-270_04.3.2). Informed consent was not obtained from the patient. The breast MRI was performed on 205 consecutive female patients scheduled to undergo treatment for pathologically proven invasive ductal carcinomas from January 2019 to December 2021. Fifteen patients were excluded due to previous resection or drug therapy and radiation therapy (n = 4), lack of pathologic biomarkers (n = 5), and incomplete menstrual history information in clinical medical records (n = 6). The largest tumor was chosen for analysis from patients with multiple synchronous tumors in the same breast. Finally, this study included 190 patients with 190 lesions (**Figure 1**). In addition, we used clinical information-based variables such as patient age, menstrual status, and tumor TIC type in this study.

### MRI acquisition and analysis

A 3-T MRI system (American GE Singa HDxt) and a dedicated eight-channel breast coil were used to perform the MRI in the prone position (NORAS MRI products). A total of nine phases were scanned, and the first phase was a plain scan mask. The plain scan included: (1) cross-sectional fat-suppressed T2 sequence (TR 3550 ms, TE 102 ms); (2) cross-sectional T1 sequence (TR 4.4 ms, TE 2.1 ms); (3) cross-sectional diffusion-weighted imaging (DWI) sequence (TR 6000ms, TE 69.2ms), slice thickness/slice spacing were both 4.0 mm/1.0 ms; and(4) dynamic enhancement was performed with cross-sectional Vibrant + enhancement sequence (TR 4.4 ms, TE 2.1 ms, layer thickness 1.2 mm). Gadopentetate meglumine (Gd-DTPA) was rapidly injected through the dorsal vein of the hand with a high-pressure syringe at a bolus injection rate of 2.0 ml/s and a dose of 0.2 ml/kg, followed by a rapid bolus injection of 20 mL of normal saline. Eight dynamic enhancement sequences were continuously scanned at 61s, 122s, 184s, 245s, 306s, 368s, and 429s after the injection.

**Figure 2:**
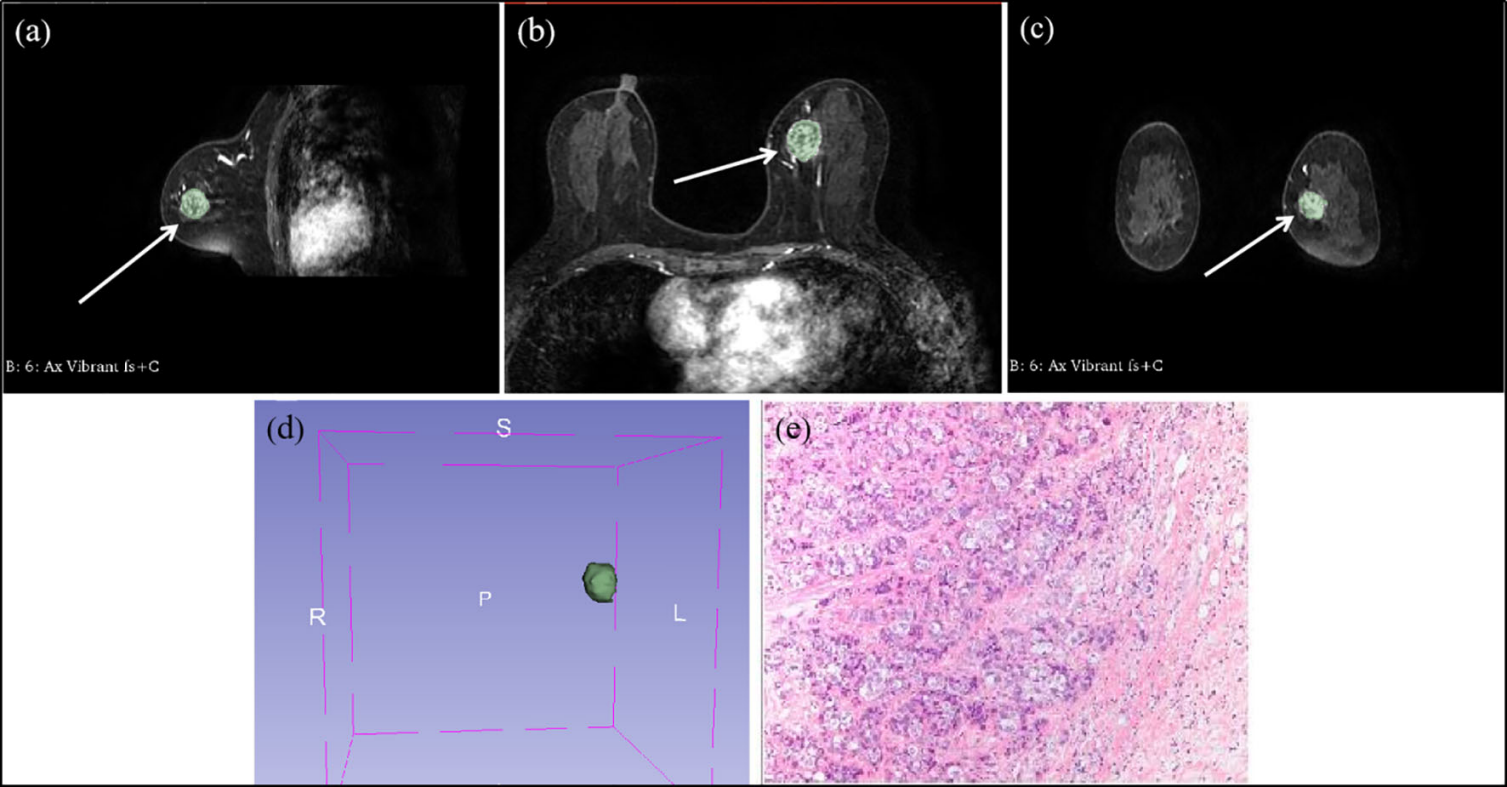
Multi-level step-by-step sketching of tumor lesions at contrast-enhanced T1-weighted MRI in a 49 year-old woman with invasive ductal cancer of the left breast. **(A)** sagittal position image shows an irregular shaped, irregular margined, heterogeneous enhancing mass (arrow). **(B)** transverse position image shows an irregular shaped, irregular margined, heterogeneous enhancing mass (arrow). **(C)** coronal position image shows an irregular shaped, irregular margined, heterogeneous enhancing mass (arrow). **(D)** the VOI fused into the outlined images step-by-step. **(E)** the pathological microscopic picture of invasive ductal cancer of the left breast.

Two radiologists (L.Z.Y. and F.G., with 10 and 5 years of experience in breast MRI, respectively) were blinded and evaluated MRI features in consensus. Multi-level step-by-step sketching of tumor lesions in T1 images of stage 2 after contrast injection using commercially available image processing software 3D Slicer (https://www.slicer.org, version number: 4.11.20210226 r29738/7a593c8). The sketched images were finally fused to form a three-dimensional VOI (Volume of Interest), as shown in **Figure 2**. Image features such as shape-based, first-order statistical, texture, wavelet, and laplacian of Gaussian (LOG) filter were extracted.

### Pathologic assessment

The molecular subtypes of breast cancers in this study were classified based on the expert consensus of the 2013 St. Gallen International Breast Cancer Conference (24) as follows: Luminal A (ER+, HER2–, and Ki67–); Luminal B (ER+, HER2–, and Ki67+; or ER+, HER2+, and Ki67); HER2-overexpressed (ER–, PR–, and HER2+); and triple-negative cancer (ER–, PR–, and HER2–). From a total of 190 patients, a mastectomy was performed on 60 patients, breast-conservation surgery was performed on 125 patients, and neoadjuvant chemotherapy and surgery were performed on five patients. We reviewed the pathology reports and included tumor size, histological grade, and axillary lymph node metastases in the statistical analysis.

### Statistical analysis and model evaluation

This study divided all of the enrolled patients into three categories based on postoperative immunohistochemical molecular subtypes: (a). “luminal type”, (b). “HER2-overexpressed type” and (c). “triple-negative type”. For general data processing, analysis, and related graphics of the two groups under different categories, SAS 9.4 software and R language 3.6.1 (http://www.R-project.org) tools are used. Measurement data were expressed as mean ± standard deviation. The frequency data expressed the count data (composition ratio, percent). A t-test was used to figure out the age. For the largest diameter, the rank-sum test was used. The chi-square test determined menstrual status and axillary lymph node metastasis. The nonparametric Mann Whitney U test determined the tumor’s histological grade and TIC.

**Figure 3:**
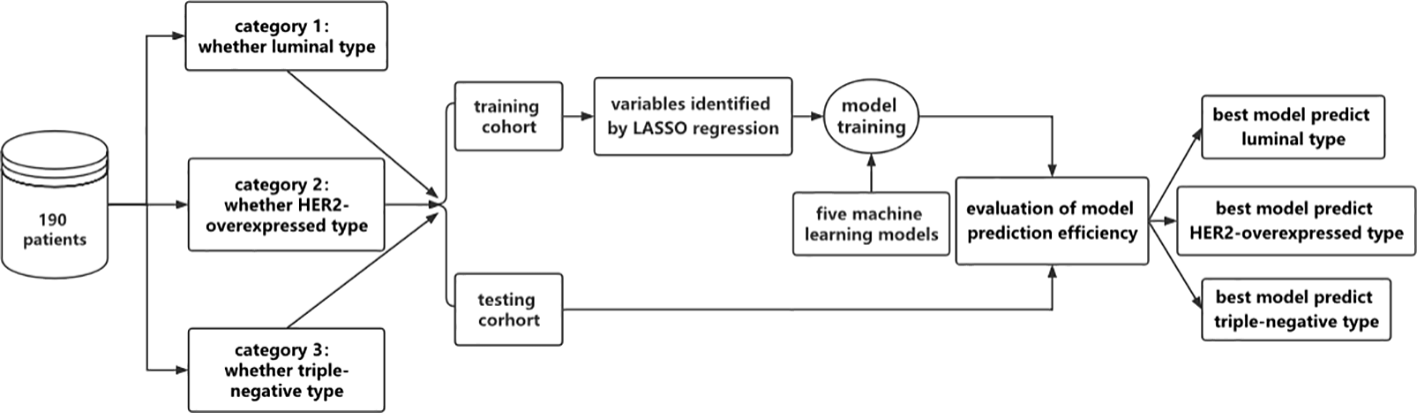
Flow chart of model establishment in this study. The 190 patients were grouped according to different molecular pressure types and divided into 3 categories, namely luminal and non-luminal, HER2-overexpressed and non-HER2-overexpressed, triple negative and non-triple negative. The following data were divided into training dataset and testing dataset. In the training dataset, the feature variables were screened by LASSO regression, and five machine learning models were used to construct the model. The performance evaluation of the model was carried out in the testing dataset to determine the optimal model.

Using the R language random grouping function, the sample data for each category standard were randomly divided into the model training cohort and the model validation cohort in a 7:3 ratio. The pathology report variables and the MRI imaging characteristics parameters were both entered into the selection process, as shown in **Figure 3**. Least Absolute Shrinkage and Selection Operator (LASSO) regression was used to avoid the potential collinearity of variables measured from the same patient and over-fitting variables. Based on the value of λ, this logistic regression model penalizes the absolute size of the coefficients of a regression model. The estimates of weaker factors shrink toward zero as the penalties become more significant, leaving only the strongest predictors in the model. The optimal λ was used to select the most predictive covariates. Following that, we looked at five machine learning models for predicting molecular subtypes based on variables determined by LASSO regression: logistic regression (LR), support vector machine (SVM), naïve Bayes (NB), random forest (RF), and eXtreme Gradient Boosting (XGBoost). To verify the classification accuracy, a 5-fold cross-validation method was used to randomly divide the entire sample into five groups. In each round, four of the groups were used as a training set and one served as a validation set. This process was repeated five times until each group of the sample had been verified, and the mean accuracy, sensitivity, specificity, AUC, F1-Score, precision, geometric mean (GM), and false positive rate (FPR) of the training sets were calculated. The confusion matrix between the actual value and the prediction value of all samples was also calculated to make a comprehensive evaluation of the model. The parameters were defined as follows:

Accuracy = (True positive + True negative)/(True positive + True negative + False positive + False negative)
Sensitivity (Recall) = True positive/(True positive + False negative)
Specificity = True negative/(False positive + True negative)
FPR = False positive/(False positive + True negative)
Precision = True positive/(True positive + False positive)
F1-Score = 2 (Recall * Precision)/(Recall + Precision)
Geometric Mean = (Recall * Specificity) ^ 1/2

Each model’s median value was calculated after the random split, and the analysis was repeated five times. Two statisticians (L.Z.Y. and S.Q.K.) conducted the analyses in January 2022.

## Results

### Patient characteristics

This study included 190 patients with bulky breast cancers (mean age 48.67 years; age range 24–89 years). The mean diameter of the breast tumors was 35.29 ± 24.23 mm. There were 99 cases of Luminal type, 59 cases were HER2-overexpressed, and 32 cases were Triple-negative type. The tumor characteristics were shown in **Table 1**.

**Table 1:** Tumor characteristics.

Characteristics	No. of breast cancers (n = 190)
Age (year) (mean ± sd)	48.67 ± 10.03
Menstrual status	
menopause	77 (40.53)
no menopause	113 (59.47)
Tumor size (mm) (mean ± sd)	35.29 ± 24.23 mm
Tumor histological grade	
I	51 (26.84)
II	95 (50.00)
III	44 (23.16)
TIC type	
I	2 (1.05)
II	68 (35.79)
III	120 (63.16)
Axillary lymph node metastases	
yes	113 (59.47)
no	77 (40.53)
Molecular subtype	
Category 1	
Luminal (A+B)	99 (52.11)
Non-luminal	91 (47.89)
Category 2	
HER2-overexpressed	59 (31.05)
Non-HER2-overexpressed	131 (68.95)
Category 3	
Triple-negative type	32 (16.84)
Non-triple-negative type	158 (83.16)

TIC, Time Intensity Curve.

### Feature selection

#### Category 1: luminal (A+B) *vs.* non-luminal

The 190 patients were divided into Luminal and Non-Luminal groups in “Luminal *vs*. Non-luminal groups”. The general characteristics of the two groups of patients are shown in **Supplemental Table 1**. The Luminal type group includes 99 patients (ages 24 to 77, with an average of 49.17 ± 10.36 years old), with 34 cases of Luminal A-type and 65 cases of Luminal B type. In the non-Luminal group, there were 91 cases (ages ranged from 29 to 89 years, with a mean of 48.54 ± 9.70 years).

**Figure 4:**
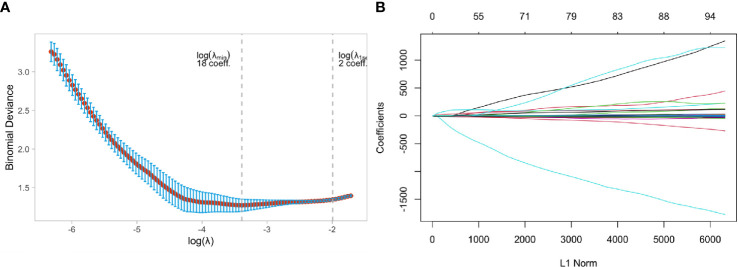
Feature selection using the least absolute shrinkage and selection operator (LASSO) binary logistic regression model in Luminal and Non-luminal group. **(A)** Tuning parameter selection in the LASSO model used 5-fold cross-validation via minimum criteria, **(B)** LASSO coefficient profiles of the baseline features.

Two independent samples t-tests and LASSO regression in R language were used to screen 1130 MRI radiomics features and five clinical features. **Figure 4A** showed a relatively stable model ability when the number of screened feature variables was 18, and the tuning parameter (λ) selection was 0.05847632. The eigenvalues vary with the value of different variables, as shown in **Figure 4B**. Finally, eighteen features with non-zero coefficients (13 wavelet transform features, five LOG features) were identified, and their coefficients were shown in **Supplemental Figure 1**.

1. log.sigma.1.0.mm.3D.firstorder.Maximum
2. log.sigma.3.0.mm.3D.glcm.Imc1
3. log.sigma.3.0.mm.3D.gldm.LargeDependenceEmphasis
4. log.sigma.3.0.mm.3D.glrlm.ShortRunEmphasis
5. log.sigma.5.0.mm.3D.firstorder.Maximum
6. wavelet.LLH.glcm.Correlation
7. wavelet.LLH.glcm.MCC
8. wavelet.LHL.firstorder.Mean
9. wavelet.LHH.glcm.JointAverage
10. wavelet.LHH.glcm.SumAverage
11. wavelet.LHH.gldm.SmallDependenceHighGrayLevelEmphasis
12. wavelet.LHH.gldm.SmallDependenceLowGrayLevelEmphasis
13. wavelet.LHH.glszm.SizeZoneNonUniformityNormalized
14. wavelet.HLH.glcm.ClusterProminence
15. wavelet.HLH.glszm.SizeZoneNonUniformityNormalized
16. wavelet.HHL.firstorder.Kurtosis
17. wavelet.HHL.firstorder.Maximum
18. wavelet.HHL.firstorder.Mean

#### Category 2: HER2-overexpressed *vs.* non-HER2-overexpressed

A total of 190 patients were divided into HER-2 overexpressed and Non-HER-2 overexpressed groups in the category “HER-2 overexpressed *vs*. Non-HER-2 overexpressed groups”. The general characteristics of the two groups of patients are shown in **Supplemental Table 2**. There were 59 cases in the HER2-overexpressed group (ages 29 to 75, mean age 48.47 ± 9.31 years old) and 131 cases in the non-HER2-overexpressed group (ages 24 to 89, mean age 48.75 ± 10.33 years old).

**Figure 5:**
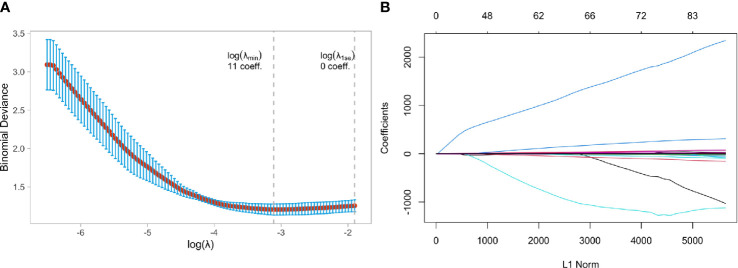
Feature selection using the least absolute shrinkage and selection operator (LASSO) binary logistic regression model in HER2-overexpressed and Non-HER2-overexpressed group. **(A)** Tuning parameter selection in the LASSO model used 5-fold cross-validation via minimum criteria, **(B)** LASSO coefficient profiles of the baseline features.

**Figure 5A** showed that the model ability is relatively stable when the number of selected feature variables was 11, and the tuning parameter (λ) selection was 0.07596331. The eigenvalues vary with the value of different variables, as shown in **Figure 5B**. Finally, eleven non-zero coefficient features (5 wavelet features, three morphological features, one texture feature, and two LOG features) were determined, and their coefficients were shown in **Supplemental Figure 2**.

1. original.shape.Maximum2DDiameterColumn
2. original.shape.Maximum2DDiameterSlice
3. original.shape.Sphericity
4. original.glcm.Imc2
5. log.sigma.3.0.mm.3D.glrlm.ShortRunEmphasis
6. log.sigma.5.0.mm.3D.firstorder.Maximum
7. wavelet.LLH.glcm.Correlation
8. wavelet.LHH.gldm.SmallDependenceHighGrayLevelEmphasis
9. wavelet.LHH.glszm.SizeZoneNonUniformityNormalized
10. wavelet.LLL.gldm.LargeDependenceHighGrayLevelEmphasis
11. wavelet.LLL.glrlm.LongRunHighGrayLevelEmphasis

#### Category 3: triple-negative type *vs.* non-triple-negative type

In the category “Triple-negative *vs*. Non-triple-negative type”, 190 patients were split into triple-negative and non-triple-negative types. The general characteristics of the two groups of patients are shown in **Supplemental Table 3**. The triple-negative type group had 32 cases (range of 30 to 77 years old, mean age 48.91 ± 10.89 years), and the non-triple-negative type group had 158 cases (range of 24 to 77, mean age 48.62 ± 9.84 years).

**Figure 6:**
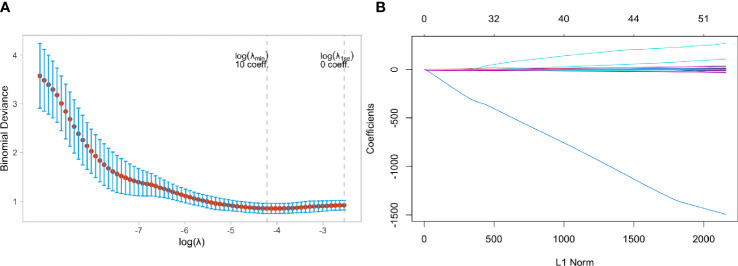
Feature selection using the least absolute shrinkage and selection operator (LASSO) binary logistic regression model in Triple negative and Non-Triple negative group. **(A)** Tuning parameter selection in the LASSO model used 5-fold cross-validation via minimum criteria, **(B)** LASSO coefficient profiles of the baseline features.

**Figure 6A** showed that the model ability is relatively stable when the number of selected feature variables was 10 and the tuning parameter (λ) selection was 0.04567723. The eigenvalues vary with the value of different variables, as shown in **Figure 6B**. Finally, ten non-zero coefficient features (8 wavelet transform features, one clinical feature, and one LOG feature) were identified, and their coefficients were shown in **Supplemental Figure 3**.

1. NA.NA.Menstrual.status
2. log.sigma.1.0.mm.3D.gldm.SmallDependenceLowGrayLevelEmphasis
3. wavelet.LHL.firstorder.Mean
4. wavelet.LHH.firstorder.Kurtosis
5. wavelet.LHH.gldm.DependenceEntropy
6. wavelet.HLL.gldm.LargeDependenceHighGrayLevelEmphasis
7. wavelet.HLH.glcm.Contrast
8. wavelet.HLH.glszm.GrayLevelVariance
9. wavelet.HHL.glcm.Correlation
10. wavelet.LLL.gldm.LargeDependenceHighGrayLevelEmphasis

### Model training and evaluation

#### Category 1: luminal (A+B) *vs.* non-luminal

**Table 2** showed the prediction performance indicators of the five machine learning models that used the variables chosen by LASSO regression to predict category 1. In the testing cohort, the XGBoost model performed the best in terms of prediction, with an AUC of 0.8282 *vs*. 0.7126 (LR), 0.7708 (RF), 0.7139 (NB), and 0.7420 (SVM). In the testing cohort, the XGBoost model’s sensitivity and specificity were 0.7524 and 0.6542, respectively, compared to 0.5556 and 0.7241 for LR, 0.7931 and 0.4815 for RF,0.3333 and 0.8276 for NB, and 0.5926 and 0.7931 for SVM. **Supplemental Table 4 ~Table 11** showed the average evaluation measures of the 5-fold cross-validation.

**Table 2:** Evaluation indicators of predictive performance of five models in Luminal group and Non-luminal group.

Classifier		SEN	SPE	PRE	GM	FPR	F1	ACC	AUC
LR	Training Dataset	0.7524	0.7052	0.7293	0.7311	0.2948	0.7383	0.7282	0.7926
	Testing Dataset	0.5556	0.7241	0.6522	0.6343	0.2759	0.6343	0.6429	0.7126
RF	Training Dataset	0.8808	0.5895	0.6953	0.7163	0.4105	0.7759	0.7384	0.8523
	Testing Dataset	0.7931	0.4815	0.6216	0.6180	0.5185	0.6180	0.6429	0.7708
NB	Training Dataset	0.8907	0.4421	0.6277	0.6294	0.5579	0.7359	0.6717	0.7884
	Testing Dataset	0.3333	0.8276	0.6429	0.5252	0.1724	0.5252	0.5893	0.7139
SVM	Training Dataset	0.8427	0.6842	0.7422	0.7571	0.3158	0.7863	0.7641	0.8626
	Testing Dataset	0.5926	0.7931	0.7273	0.6856	0.2069	0.6856	0.6964	0.7420
XGBoost	Training Dataset	0.9571	0.5895	0.7139	0.7520	0.4105	0.8223	0.7846	0.9242
	Testing Dataset	0.7524	0.6542	0.8524	0.7016	0.3458	0.6086	0.6964	0.8282

LR, Logistic Regression; RF, Random Forest; NB, Naïve Bayes, SVM, Support Vector Machine; XGBoost, eXtreme Gradient Boosting; SEN, sensitivity; SPE, specificity, PRE, precision; GM, geometric mean; FPR, false positive rate; ACC, accuracy; AUC, area under ROC.

#### Category 2: HER2-overexpressed *vs.* non-HER2-overexpressed

**Table 3** showed the prediction performance indicators of the five machine learning models that used Lasso regression to select variables to predict category 2. In the testing cohort,the RF model performed best for prediction, with an AUC of 0.8054 vs. 0.7029 (LR),0.7164 (NB), 0.7617 (SVM), and 0.7459 (XGBoost). In the testing cohort,the RF model’s sensitivity and specificity were 0.2941 and 0.9744, respectively, compared to 0.8462 and 0.3529 for LR, 0.8718 and 0.3529 for NB, 0.8974 and 0.4118 for SVM, and 0.7949 and 0.6471 for XGBoost. The average sensitivity, specificity, FPR, F1-Score, and geometric mean for the 5-fold cross-validation are shown in **Supplemental Table 12~Table 19**.

**Table 3:** Evaluation indicators of predictive performance of five models in HER2-overexpressed and Non-HER2-overexpressed groups.

Classifier		SEN	SPE	PRE	GM	FPR	F1	ACC	AUC
LR	Training Dataset	0.5000	0.7926	0.5232	0.5744	0.2074	0.5067	0.7026	0.7068
	Testing Dataset	0.8462	0.3529	0.7500	0.5465	0.6471	0.7952	0.6964	0.7029
RF	Training Dataset	0.3667	0.9704	0.9449	0.5782	0.0296	0.5105	0.7862	0.8065
	Testing Dataset	0.2941	0.9744	0.8333	0.5353	0.0256	0.4348	0.7679	0.8054
NB	Training Dataset	0.3833	0.8296	0.5958	0.5338	0.1704	0.4308	0.6923	0.6932
	Testing Dataset	0.8718	0.3529	0.7556	0.5547	0.6471	0.8095	0.7143	0.7164
SVM	Training Dataset	0.3667	0.8963	0.8269	0.5580	0.1037	0.4563	0.7333	0.7883
	Testing Dataset	0.8974	0.4118	0.7778	0.6079	0.5882	0.8333	0.7500	0.7617
XGBoost	Training Dataset	0.5167	0.9185	0.7972	0.6341	0.0815	0.6049	0.7949	0.7988
	Testing Dataset	0.7949	0.6471	0.8378	0.7172	0.3529	0.8158	0.7500	0.7459

RF, Random Forest; NB, Naïve Bayes; SVM, Support Vector Machine; XGBoost, eXtreme Gradient Boosting; SEN, sensitivity ;SPE, specificity; PRE, precision; GM, geometric mean; FPR, false positive rate; ACC, accuracy; AUC, area under ROC. LR, Logistic Regression.

#### Category 3: triple-negative type *vs.* non-triple-negative type

**Table 4** showed the prediction performance indicators of the five machine learning models that used Lasso regression to select variables to predict category 3. In the testing cohort, the XGBoost model performed the best in terms of prediction, with an AUC of 0.9031 vs. 0.7069(LR), 0.7979(RF), 0.6809(NB), and 0.7778(SVM). In the testing cohort, the XGBoost model’s sensitivity and specificity were 0.9362 and 0.4444, respectively, compared to 0.9149 and 0.3333 for LR, 0.1111 and 1.0000 for RF, 0. 8723 and 0.3333 for NB, and 1.0000 and 0.2222 for SVM. The average sensitivity, specificity, FPR, F1-Score, and geometric mean for the 5-fold cross-validation are shown in **Supplemental Table 20 ~ Table 27**.

**Table 4:** Evaluation indicators of predictive performance of five models in Triple-negative group and Non-triple-negative group.

Classifier		SEN	SPE	PRE	GM	FPR	F1	ACC	AUC
LR	Training Dataset	0.3043	0.9459	0.5344	0.7143	0.0541	0.3867	0.8358	0.7773
	Testing Dataset	0.9149	0.3333	0.8776	0.5522	0.6667	0.8958	0.8214	0.7069
RF	Training Dataset	0.1217	1.0000	1.0000	0.5958	0	0.2156	0.8493	0.8722
	Testing Dataset	0.1111	1.0000	1.0000	0.3333	0	0.2000	0.8571	0.7979
NB	Training Dataset	0.3217	0.9027	0.4068	0.7057	0.0973	0.3576	0.8030	0.7451
	Testing Dataset	0.8723	0.3333	0.8723	0.5392	0.6667	0.8723	0.7857	0.6809
SVM	Training Dataset	0.1565	1.0000	1.0000	0.6069	0	0.2691	0.8552	0.8743
	Testing Dataset	1.0000	0.2222	0.8704	0.4714	0.7778	0.9307	0.8750	0.7778
XGBoost	Training Dataset	0.4000	0.9730	0.7555	0.7669	0.027	0.5210	0.8746	0.9260
	Testing Dataset	0.9362	0.4444	0.8980	0.6450	0.5556	0.9167	0.8571	0.9031

LR, Logistic Regression; RF, Random Forest; NB, Naïve Bayes; SVM, Support Vector Machine; XGBoost, eXtreme Gradient Boosting; SEN, sensitivity; SPE, specificity; PRE, precision; GM, geometric mean; FPR, false positive rate; ACC, accuracy; AUC. area under ROC.

## Discussion

Our research found that combining MRI radiomic variables, pathology variables, and clinical data could help predict invasive ductal breast cancer molecular subtypes. The XGBoost method outperformed the other five machine learning models in predicting luminal and triple-negative types. In the HER2-overexpressed type, the RF method had the best predictive performance. We believe that developing an MRI-based diagnosis prediction model can provide a unique idea for clinical non-invasive prediction of breast cancer molecular subtype classification and a benchmark for developing clinically precise and individualized treatment plans from our findings.

We extracted five categories features from DCE-MRI: shape-based features, first-order statistical features, texture features, wavelet features, and the laplacian of a gaussian filter. Wavelet features can be used to calculate image signal resolution on various temporal, spatial, and frequency scale planes (25). Texture analysis extracts and quantifies information such as regularity, roughness, and the grey level of lesions that cannot be distinguished by the naked eye, allowing for a more comprehensive and detailed reflection of the characteristics of lesions (26). Texture analysis plays a vital role in molecular typing and can effectively distinguish between HR-positive and HR-negative breast cancers (26). Three radiological features were extracted by Tagliafico et al. (27): energy, entropy, and difference. There were significant differences between breast and normal breast tissue in patients with dense breasts. This research shows that radiomics has much potential for detecting malignant features in breast lesions.

Three first-order statistical features (minimum value, average value, and maximum value) were selected in the luminal group. Two first-order statistical features (maximum and average) were selected in the HER2-overexpressed group. Two first-order statistical features (kurtosis and mean value) were selected in the triple-negative group in our study. Ming Fan et al. (28) found that luminal A had low kurtosis and skewness, the essential features in predictive models. This finding is in line with previous research suggesting that skewness can be used to predict breast cancer molecular subtypes (11). Kurtosis and skewness signatures have been identified as biomarkers of tumor heterogeneity (29), with high values indicating treatment failure (30) and low values indicating treatment response (31). These studies discovered differences in parenchymal background enhancement between normal and abnormal breasts. These differences could reflect the aggressiveness of breast tumors, which is one of the main characteristics of the HER2-overexpressed type. Triple-negative breast cancer cells are more disordered, loose, and prone to necrosis (32), and HER2-overexpressed breast cancer behaves more like triple-negative breast cancer, likely due to less aggressive tumors’ lower neovascularization (33). This study chose no related first-order features in the luminal and HER2-overexpressed groups. We believe this is related to the sample size and the grouping method. The kurtosis was chosen in the triple-negative group, which could be due to tumor heterogeneity.

Building predictive models and model selection are critical in radiomics to ensure reliability and stability (34–39). In both luminal and non-luminal groups, the XGBoost model outperforms the other five models. The model’s AUC in the validation cohort was 0.8282, indicating that it was more efficient at classification. The sensitivity was 0.7524, indicating that positive samples could be detected on average. In terms of prediction, previous research has shown that the XGBoost model outperforms other machine learning algorithms (40–43). Our findings were comparable to or better than previous retrospective radiomic studies on breast MRI (44, 45). Aside from that, the triple-negative and non-triple-negative groups had similar outcomes. Although some studies have found that logistic regression is effective in the radiomic diagnostic prediction model of triple-negative type breast cancer (22, 46), we believe this is due to differences in the number of patients in the sample and the MRI radiomic characteristics used in the studies. In our study, there were only 32 triple-negative patients.

In both the HER2-overexpressed and non-HER2-overexpressed groups, the RF model outperformed the other five models. The model’s AUC in the validation cohort was 0.8054, indicating it was more efficient at classification. The model’s specificity was 0.9744 indicating that it could, on average, distinguish negative samples. In contrast to the literature, Ma et al. (21) discovered that the RF model could distinguish HER2-expressed breast cancer by extracting radiological features from digital mammography images. Between the HER2-overexpressed and non-HER2-overexpressed groups, the AUC difference was 0.784. The AUC under the RF model was similar in our study than in Ma’s. The intrinsic differences between two-dimensional mammogram images and three-dimensional DCE-MRI images, we believe, are to blame. MRI can reveal details that digital mammography cannot, such as the location, size, morphology, surrounding tissue infiltration, intratumoral hemorrhage, necrosis, etc. Breast MRI was used by Braman et al. (23) to extract radiological features around and within the tumor, demonstrating that similar features can identify HER2-overexpressed breast cancer.

According to previous research, the incidence rates of luminal type, HER2-overexpressed type, and triple-negative breast cancer were 44.5% - 69.0%, 10% - 25%, and 10% - 20%, respectively (46). Our study’s incidence of these three types of breast cancer supports this theory. They are 52.78% for the luminal type, 32.41% for the HER2-overexpressed type, and 14.81% for the HER2-overexpressed type (triple-negative type). Our research is one of the few MRI diagnostic models for breast cancer that includes all three types of cancer and is based on many patient samples. Small sample sizes of around 100 were used in most radiomic studies of breast cancer, resulting in selection bias and affecting the results (47). For obtaining uniform results and building predictive models in radiomics, collecting methods, sample size, and imaging acquisition are critical. Second, we used commercially available image analysis software to create a three-dimensional image of the tumor (48), ensuring that more relevant variables were captured. It may be possible to conduct multicenter external validation studies to measure the predictive performance of radiomic machine learning models objectively. Most radiomics have been used in scientific research, but they are not widely used in clinics. Third, we identified key MRI parameters for predicting prognostic factors in various breast cancer subtypes, primarily molecular subtypes. The advancement of precision medicine in breast cancer will be aided by developing more detailed and precise assays for the important parameters discovered in our study.

In terms of clinical application, our findings show that additional information can be added before and after treatment, in addition to pathological correlations. Clinical biomarkers for prognosis prediction and treatment monitoring can be created by combining important MRI radiomic and pathology variables. They reflect microstructural tumor characteristics like tumor heterogeneity and angiogenesis, allowing for noninvasive intratumoral dynamics monitoring throughout treatment.

Texture heterogeneity was discovered by Trebeschi et al. (49) to be a noninvasive imaging biomarker for predicting immunotherapy responses that could be used in neoadjuvant and palliative settings. Our findings show that radiomics has the potential to improve patient stratification and treatment planning.

Lubner et al. (48) found that the results captured from two-dimensional and three-dimensional image features in untreated hepatic metastatic colorectal cancer were comparable. Our results were slightly better than those of the most recent study (20), which used two-dimensional images for feature selection, with an AUC of 0.83 *vs*. 0.80 in the lumina and non-luminal groups, and 0.81 *vs*. 0.65 in the HER2-overexpressed and non-HER2-overexpressed groups, suggesting that three-dimensional images may have advantages.

There are some limitations to our study. First, this study did not assess the reproducibility of segmentation for image analysis. To avoid problems with lesion selection, our study had two experienced radiologists evaluate MRI features blinded to the clinicopathologic findings. Second, for internal validation, the random split and analysis were repeated five times, and the average AUCs for each machine learning model in this study were calculated. The validity of this study is expected to improve with more random splits and analyses on internal and external data sets, such as 50 times. Third, we did not include other sequences due to the practical advantage of quick acquisition times (50–52). This study obtained a cross-section fat-suppressed T2 sequence, cross-sectional T1 sequence, cross-sectional DWI sequence, and cross-sectional Vibrant + enhancement sequence.

Recent research has found a strong link between quantitative MRI parameters and breast cancer aggressiveness and subtypes in ultrafast and diffusion-weighted MRI (50–52). A multi-center clinical prospective study with larger patient samples and more MRI sequence features extracted currently underway. We are excited to learn more about the results of this long-term study and we look forward to enrolling more patients and observing such results in our next phase of clinical research.

## Conclusion

Our research found promising results from a radiomic machine learning analysis that combined pathology variables, clinical information, and radiomic variables on MRI to achieve noninvasive and objective diagnostic factor prediction for different molecular subtypes of invasive ductal breast cancer. The RF model had the best predictive performance for distinguishing HER2-overexpressed types, while the XGBoost model showed the best predictive performance for distinguishing luminal and triple-negative types.

## Data availability statement

The raw data supporting the conclusions of this article will be made available by the authors, without undue reservation.

## Ethics statement

The studies involving human participants were reviewed and approved by the ethics committee of the First Affiliated Hospital of Guangzhou University of Chinese Medicine (ethics approval number: JY2021-270_04.3.2). Written informed consent for participation was not required for this study in accordance with the national legislation and the institutional requirements.

## Author contributions

SX and YZ contributed the study design, FG and YW contribute the data acquisition, EM for the language polish, WS for the valuable comments on the article review. We thank LY and SK for the data analysis. We thank TT for helping contact all members of the team for early collaboration. All authors contributed to the article and approved the submitted version.

## Acknowledgments

We thank Professor Gao Xuan for his writing guidance for this article.

## Conflict of interest

The authors declare that the research was conducted in the absence of any commercial or financial relationships that could be construed as a potential conflict of interest.

## Publisher’s note

All claims expressed in this article are solely those of the authors and do not necessarily represent those of their affiliated organizations, or those of the publisher, the editors and the reviewers. Any product that may be evaluated in this article, or claim that may be made by its manufacturer, is not guaranteed or endorsed by the publisher.
