# Supplementary material for: Invasive ductal breast cancer molecular subtype prediction by MRI radiomic and clinical features based on machine learning
Source: Front Oncol. 2022 Sep 12;12:964605. doi: 10.3389/fonc.2022.964605 (PMC9510620; doi:10.3389/fonc.2022.964605)
Supplement: Supplementary file 1 [file DataSheet_1.docx]

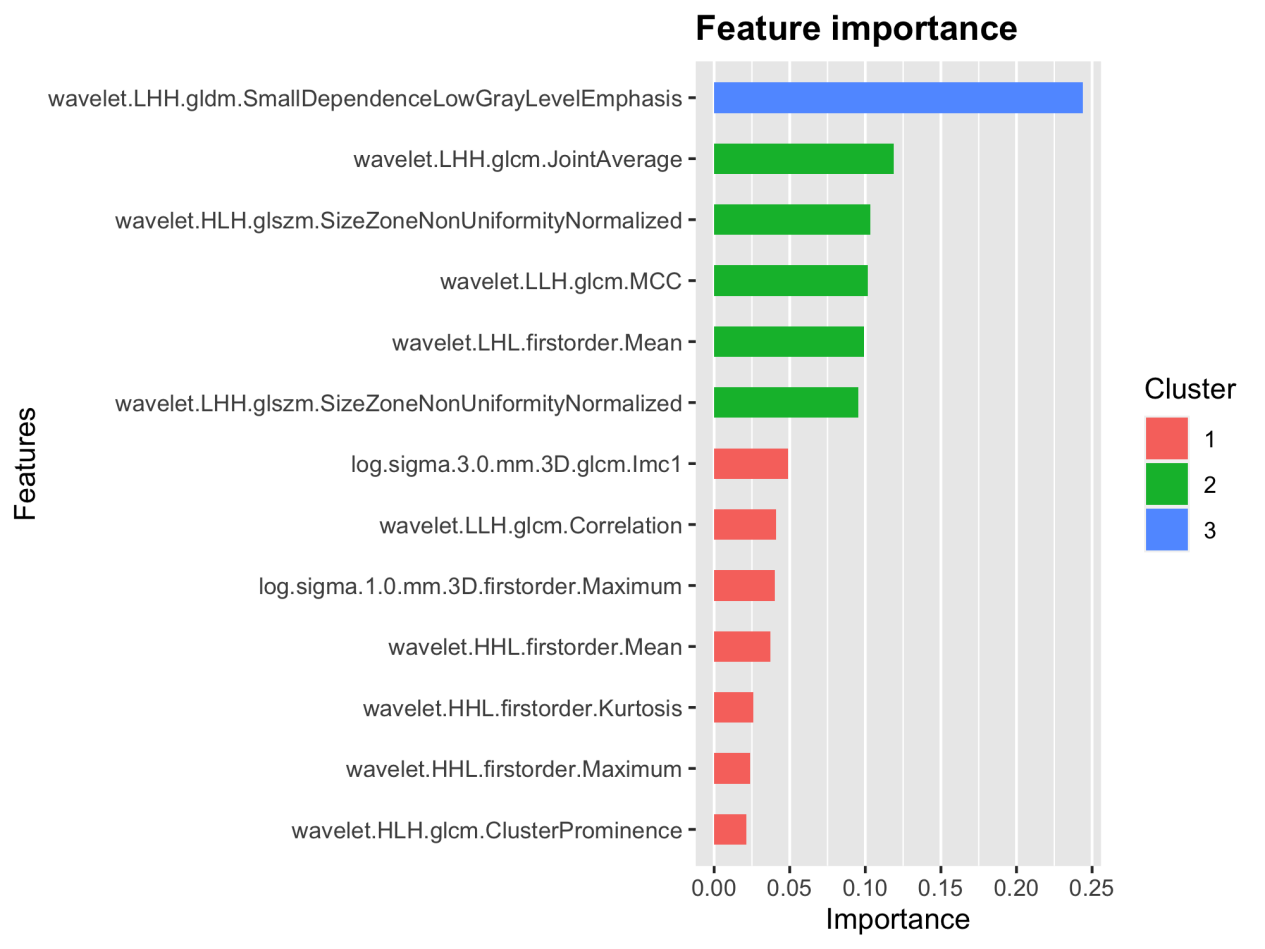


**Supplemental Figure 1: Selected feature variables and their coefficient in Luminal and Non-luminal group. The Y-axis depicts the specific feature, while the X-axis depicts the feature's corresponding LASSO coefficient. (Features that account for less than 1% are not displayed).**

**
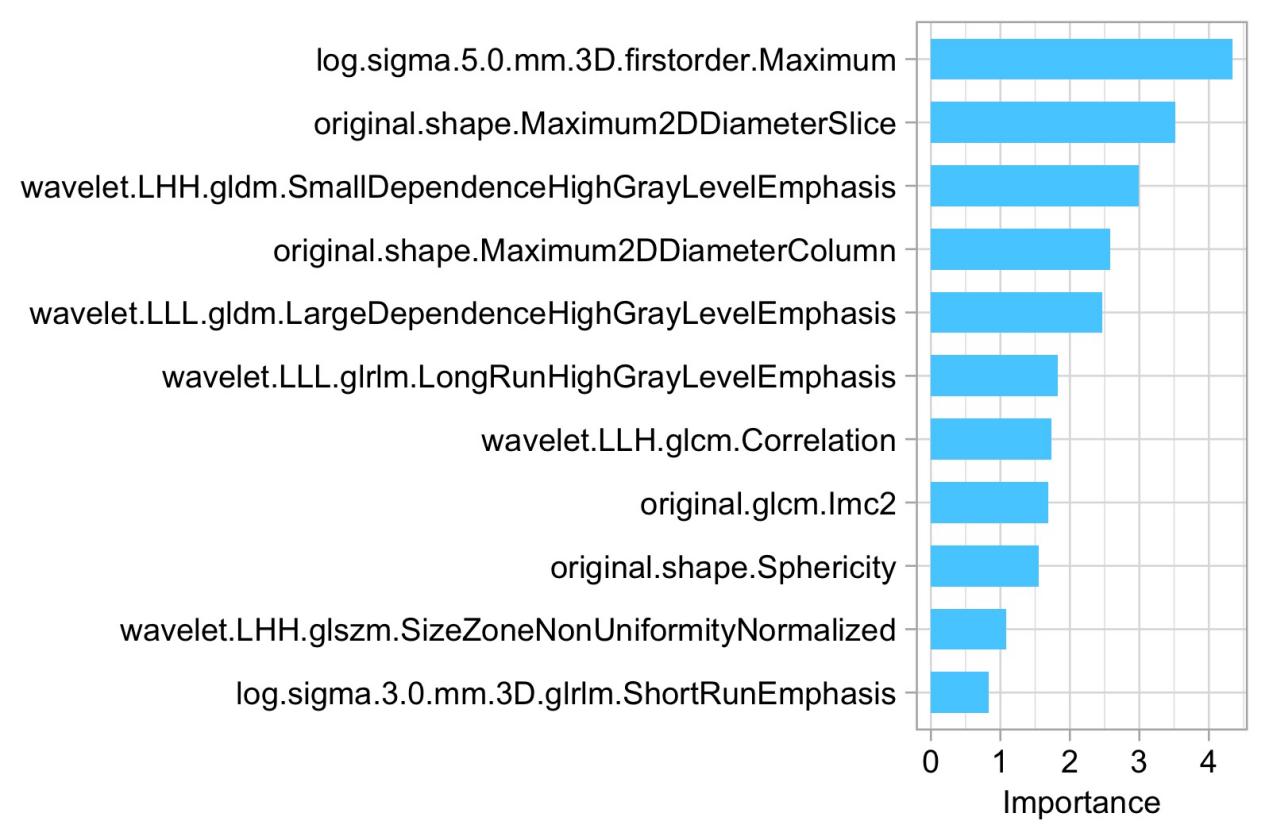
**

**Supplemental Figure 2: Selected feature variables and their coefficient in HER2-overexpressed and Non-HER2-overexpressed group. The Y-axis depicts the specific feature, while the X-axis depicts the feature's corresponding LASSO coefficient.**

**
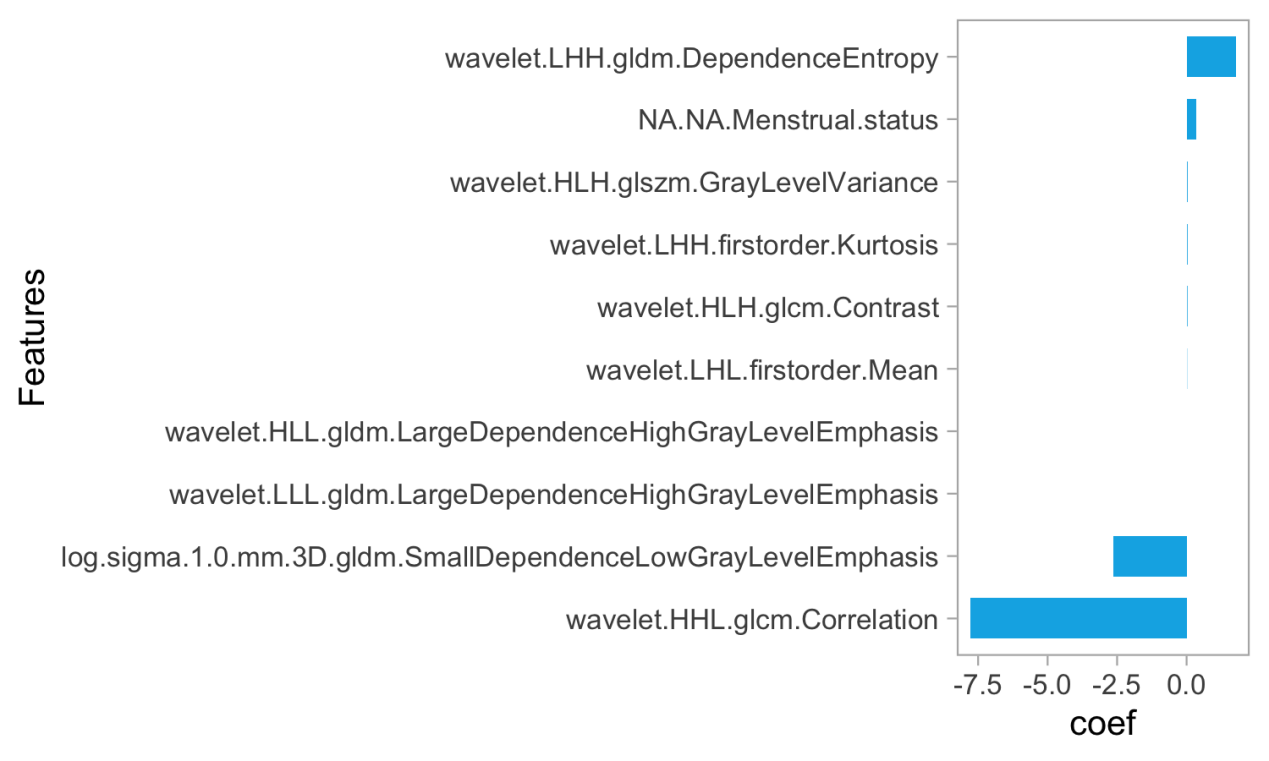
**

**Supplemental Figure 3: Selected feature variables and their coefficient in Triple-negative and Non-triple-negative type. NA.NA.p = menopause.The Y-axis depicts the specific feature, while the X-axis depicts the feature's corresponding LASSO coefficient.**
