# Supplementary material for: Invasive ductal breast cancer molecular subtype prediction by MRI radiomic and clinical features based on machine learning
Source: Front Oncol. 2022 Sep 12;12:964605. doi: 10.3389/fonc.2022.964605 (PMC9510620; doi:10.3389/fonc.2022.964605)
Supplement: Supplementary file 2 [file DataSheet_2.docx]

**Supplemental Table**

**Supplemental Table 1. Characteristics of Luminal type & Non-luminal type.**

| Characteristics | Luminal Type  n = 99 (52.11 %) | Non-luminal Type  n = 91 (47.89 %) | *P* |
| --- | --- | --- | --- |
| Age (year) (mean ± sd) | 49.17 ± 10.36 | 48.54 ± 9.70 | 0.5014 |
| Menstrual status |  | |  |
| menopause | 64 (64.65) | 49 (53.85) | 0.2714 |
| no menopause | 35 (35.35) | 42 (46.15) |  |
| Tumor size (mm) (mean ± sd) | 31.36 ± 22.99 | 38.47 ± 24.40 | 0.0342 |
| Tumor histological grade |  |  |  |
| Ⅰ | 33 (33.33) | 18 (19.78) | 0.0010 |
| Ⅱ | 54 (54.55) | 41 (45.05) |  |
| Ⅲ | 12 (12.12） | 32 (35.17) |  |
| TIC type |  |  |  |
| Ⅰ | 2 (2.02) | 0 | 0.7381 |
| Ⅱ | 35 (35.35) | 33 (36.26) |  |
| Ⅲ | 62 (62.63) | 58 (63.74) |  |
| Axillary lymph node metastases |  |  |  |
| yes | 40 (40.40) | 37 (40.66) | 0.9258 |
| no | 59 (59.60) | 54 (59.34) |  |

**Note.**—TIC = Time Intensity Curve.

**Supplemental Table 2. Characteristics of HER2-overexpressed type & Non-HER2-overexpressed type.**

| Characteristics | HER2-overexpressed Type  n = 59 (31.05 %) | Non-HER2-overexpressed Type  n = 131 (68.95 %) | *P* |
| --- | --- | --- | --- |
| Age (year) (mean ± sd) | 48.47 ± 9.31 | 48.75 ± 10.33 | 0.6139 |
| Menstrual status |  |  |  |
| menopause | 36 (61.02) | 77 (58.78) | 0.5863 |
| no menopause | 23 (38.98) | 54 (41.22) |  |
| Tumor size (mm) (mean ± sd) | 43.07 ± 24.73 | 31.79 ± 23.16 | 0.0130 |
| Tumor histological grade |  |  |  |
| Ⅰ | 15 (25.42) | 36 (27.48) | 0.9616 |
| Ⅱ | 30 (50.85) | 65 (49.62) |  |
| Ⅲ | 14 (23.73） | 30 (22.90) |  |
| TIC type |  |  |  |
| Ⅰ | 0 | 2 (1.53) | 0.4847 |
| Ⅱ | 23 (38.98) | 45 (34.35) |  |
| Ⅲ | 36 (61.02) | 84 (64.12) |  |
| Axillary lymph node metastases |  |  |  |
| yes | 25 (42.37) | 52 (39.69) | 0.8166 |
| no | 34 (57.63) | 79 (60.31) |  |

**Note.**—TIC = Time Intensity Curve.

**Supplemental Table 3. Characteristics of Triple-negative type & Non-triple-negative type.**

| Characteristics | TN Type  n = 32 (16.84 %) | Non-TN Type  n = 158 (83.16 %) | *P* |
| --- | --- | --- | --- |
| Age (year) (mean ± sd) | 48.91 ± 10.89 | 48.62± 9.84 | 0.9826 |
| Menstrual status |  |  |  |
| menopause | 13 (40.63) | 100 (63.29) | 0.0237 |
| no menopause | 19 (59.38) | 58 (36.71) |  |
| Tumor size (mm) (mean ± sd) | 43.07 ± 24.73 | 33.09 ± 23.63 | 0.5919 |
| Tumor histological grade |  |  |  |
| Ⅰ | 3 (9.38) | 48 (30.38) | < 0.0001 |
| Ⅱ | 11 (34.38) | 84 (53.16) |  |
| Ⅲ | 18 (56.24） | 26 (16.46) |  |
| TIC type |  |  |  |
| Ⅰ | 0 | 2 (1.27) | 0.3142 |
| Ⅱ | 10 (31.25) | 45 (28.48) |  |
| Ⅲ | 22 (68.75) | 84 (53.16) |  |
| Axillary lymph node metastases |  |  |  |
| yes | 12 (37.50) | 65 (41.14) | 0.8614 |
| no | 20 (62.50) | 93 (58.86) |  |

**Note.**—TIC = Time Intensity Curve, TN = Triple-negative.

**Supplemental Table 4. AUC for Cross-validation of Luminal type & Non-luminal type.**

| **Models** | **Fold 1** | **Fold 2** | **Fold 3** | **Fold 4** | **Fold 5** | **Mean** | **SD** |
| --- | --- | --- | --- | --- | --- | --- | --- |
| **LR** | 0.7658 | 0.8368 | 0.8263 | 0.7500 | 0.7842 | 0.7926 | 0.0377 |
| **RF** | 0.8408 | 0.8803 | 0.8487 | 0.8132 | 0.8789 | 0.8523 | 0.0281 |
| **NB** | 0.7974 | 0.8158 | 0.7711 | 0.7500 | 0.8079 | 0.7884 | 0.0273 |
| **SVM** | 0.8237 | 0.9211 | 0.8579 | 0.8237 | 0.8868 | 0.8626 | 0.0420 |
| **XGBoost** | 0.8921 | 0.9474 | 0.9382 | 0.9079 | 0.9355 | 0.9242 | 0.0232 |

**Note.**—LR=Logistic Regression, RF=Random Forest, NB= Naïve Bayes, SVM=Support Vector Machine, XGBoost=eXtreme Gradient Boosting, AUC= area under ROC.

**Supplemental Table 5. SEN for Cross-validation of Luminal type & Non-luminal type.**

| **Models** | **Fold 1** | **Fold 2** | **Fold 3** | **Fold 4** | **Fold 5** | **Mean** | **SD** |
| --- | --- | --- | --- | --- | --- | --- | --- |
| **LR** | 0.7023 | 0.8126 | 0.8132 | 0.8345 | 0.6432 | 0.7524 | 0.0750 |
| **RF** | 0.8456 | 0.9234 | 0.8424 | 0.9263 | 0.8438 | 0.8808 | 0.0397 |
| **NB** | 0.8521 | 0.9025 | 0.9015 | 0.9148 | 0.9287 | 0.8907 | 0.0259 |
| **SVM** | 0.8509 | 0.8534 | 0.8556 | 0.8423 | 0.8123 | 0.8427 | 0.0160 |
| **XGBoost** | 0.9460 | 0.9982 | 0.965 | 0.9376 | 0.9385 | 0.9571 | 0.0198 |

**Note.**—LR=Logistic Regression, RF=Random Forest, NB= Naïve Bayes, SVM=Support Vector Machine, XGBoost=eXtreme Gradient Boosting, SEN=sensitivity.

**Supplemental Table 6. SPE for Cross-validation of Luminal type & Non-luminal type**

| **Models** | **Fold 1** | **Fold 2** | **Fold 3** | **Fold 4** | **Fold 5** | **Mean** | **SD** |
| --- | --- | --- | --- | --- | --- | --- | --- |
| **LR** | 0.6315 | 0.7894 | 0.7368 | 0.6315 | 0.7368 | 0.7052 | 0.0632 |
| **RF** | 0.5263 | 0.5263 | 0.6842 | 0.5263 | 0.6842 | 0.5895 | 0.0774 |
| **NB** | 0.3684 | 0.4736 | 0.4736 | 0.3684 | 0.5263 | 0.4421 | 0.0631 |
| **SVM** | 0.6315 | 0.7894 | 0.6842 | 0.5263 | 0.7894 | 0.6842 | 0.0998 |
| **XGBoost** | 0.5789 | 0.5789 | 0.6842 | 0.5263 | 0.5789 | 0.5895 | 0.0516 |

**Note.**—LR=Logistic Regression, RF=Random Forest, NB= Naïve Bayes, SVM=Support Vector Machine, XGBoost=eXtreme Gradient Boosting, SPE=specificity.

**Supplemental Table 7. F1-Score for Cross-validation of Luminal type & Non-luminal type.**

| **Models** | **Fold 1** | **Fold 2** | **Fold 3** | **Fold 4** | **Fold 5** | **Mean** | **SD** |
| --- | --- | --- | --- | --- | --- | --- | --- |
| **LR** | 0.6829 | 0.8000 | 0.7804 | 0.7441 | 0.6842 | 0.7383 | 0.0538 |
| **RF** | 0.7391 | 0.7659 | 0.8181 | 0.7659 | 0.7906 | 0.7759 | 0.0298 |
| **NB** | 0.6938 | 0.7500 | 0.7500 | 0.7200 | 0.7659 | 0.7359 | 0.0288 |
| **SVM** | 0.7727 | 0.8292 | 0.7906 | 0.7391 | 0.8000 | 0.7863 | 0.0334 |
| **XGBoost** | 0.8085 | 0.8333 | 0.8695 | 0.7916 | 0.8085 | 0.8223 | 0.0303 |

**Note.**—LR=Logistic Regression, RF=Random Forest, NB= Naïve Bayes, SVM=Support Vector Machine, XGBoost=eXtreme Gradient Boosting.

**Supplemental Table 8. FPR for Cross-validation of Luminal type & Non-luminal type.**

| **Models** | **Fold 1** | **Fold 2** | **Fold 3** | **Fold 4** | **Fold 5** | **Mean** | **SD** |
| --- | --- | --- | --- | --- | --- | --- | --- |
| **LR** | 0.3684 | 0.2105 | 0.2632 | 0.3684 | 0.2632 | 0.2948 | 0.0632 |
| **RF** | 0.4737 | 0.4737 | 0.3158 | 0.4737 | 0.3158 | 0.4105 | 0.0774 |
| **NB** | 0.6316 | 0.5263 | 0.5263 | 0.6316 | 0.4737 | 0.5579 | 0.0632 |
| **SVM** | 0.3684 | 0.2105 | 0.3158 | 0.4737 | 0.2105 | 0.3158 | 0.0999 |
| **XGBoost** | 0.4211 | 0.4210 | 0.3158 | 0.4737 | 0.4211 | 0.4105 | 0.0516 |

**Note.**—LR=Logistic Regression, RF=Random Forest, NB= Naïve Bayes, SVM=Support Vector Machine, XGBoost=eXtreme Gradient Boosting, FPR=false positive rate.

**Supplemental Table 9. GM for Cross-validation of Luminal type & Non-luminal type.**

| **Models** | **Fold 1** | **Fold 2** | **Fold 3** | **Fold 4** | **Fold 5** | **Mean** | **SD** |
| --- | --- | --- | --- | --- | --- | --- | --- |
| **LR** | 0.6649 | 0.7947 | 0.7678 | 0.7108 | 0.6921 | 0.7261 | 0.0481 |
| **RF** | 0.6689 | 0.6882 | 0.7847 | 0.6882 | 0.7626 | 0.7185 | 0.0461 |
| **NB** | 0.5596 | 0.6529 | 0.6529 | 0.5758 | 0.6882 | 0.6259 | 0.0495 |
| **SVM** | 0.7327 | 0.8192 | 0.7626 | 0.6689 | 0.7947 | 0.7556 | 0.0523 |
| **XGBoost** | 0.7417 | 0.7609 | 0.8272 | 0.7072 | 0.7416 | 0.7557 | 0.0397 |

**Note.**—LR=Logistic Regression, RF=Random Forest, NB= Naïve Bayes, SVM=Support Vector Machine, XGBoost=eXtreme Gradient Boosting, GM=geometric mean.

**Supplemental Table 10. ACC for Cross-validation of Luminal type & Non-luminal type.**

| **Models** | **Fold 1** | **Fold 2** | **Fold 3** | **Fold 4** | **Fold 5** | **Mean** | **SD** |
| --- | --- | --- | --- | --- | --- | --- | --- |
| **LR** | 0.6667 | 0.7948 | 0.7692 | 0.7179 | 0.6923 | 0.7282 | 0.0531 |
| **RF** | 0.6923 | 0.7179 | 0.7948 | 0.7179 | 0.7692 | 0.7384 | 0.0421 |
| **NB** | 0.6153 | 0.6923 | 0.6923 | 0.6410 | 0.7179 | 0.6717 | 0.0421 |
| **SVM** | 0.7435 | 0.8205 | 0.7692 | 0.6923 | 0.7948 | 0.7641 | 0.0493 |
| **XGBoost** | 0.7692 | 0.7948 | 0.8461 | 0.7435 | 0.7692 | 0.7846 | 0.0388 |

**Note.**—LR=Logistic Regression, RF=Random Forest, NB= Naïve Bayes, SVM=Support Vector Machine, XGBoost=eXtreme Gradient Boosting, ACC=accuracy.

**Supplemental Table 11. PRE for Cross-validation of Luminal type & Non-luminal type.**

| **Models** | **Fold 1** | **Fold 2** | **Fold 3** | **Fold 4** | **Fold 5** | **Mean** | **SD** |
| --- | --- | --- | --- | --- | --- | --- | --- |
| **LR** | 0.6667 | 0.8000 | 0.7619 | 0.6957 | 0.7222 | 0.7293 | 0.0528 |
| **RF** | 0.6538 | 0.6667 | 0.7500 | 0.6667 | 0.7391 | 0.6953 | 0.0455 |
| **NB** | 0.5862 | 0.6429 | 0.6429 | 0.6000 | 0.6667 | 0.6277 | 0.0334 |
| **SVM** | 0.7083 | 0.8095 | 0.7391 | 0.6538 | 0.8000 | 0.7422 | 0.0649 |
| **XGBoost** | 0.7037 | 0.7143 | 0.7692 | 0.6786 | 0.7037 | 0.7139 | 0.0336 |

**Note.**—LR=Logistic Regression, RF=Random Forest, NB= Naïve Bayes, SVM=Support Vector Machine, XGBoost=eXtreme Gradient Boosting, PRE=precision.

**Supplemental Table 12. AUC for Cross-validation of HER2-overexpressed & Non-HER2-overexpressed type.**

| **Models** | **Fold 1** | **Fold 2** | **Fold 3** | **Fold 4** | **Fold 5** | **Mean** | **SD** |
| --- | --- | --- | --- | --- | --- | --- | --- |
| **LR** | 0.6790 | 0.6451 | 0.6759 | 0.8056 | 0.7284 | 0.7068 | 0.0628 |
| **RF** | 0.7731 | 0.8086 | 0.7515 | 0.9028 | 0.7963 | 0.8065 | 0.0582 |
| **NB** | 0.6204 | 0.6605 | 0.6821 | 0.8179 | 0.6852 | 0.6932 | 0.0743 |
| **SVM** | 0.7037 | 0.7623 | 0.7778 | 0.8858 | 0.8117 | 0.7883 | 0.0671 |
| **XGBoost** | 0.7778 | 0.7963 | 0.7145 | 0.8472 | 0.8580 | 0.7988 | 0.0579 |

**Note.**—LR=Logistic Regression, RF=Random Forest, NB= Naïve Bayes, SVM=Support Vector Machine, XGBoost=eXtreme Gradient Boosting, AUC= area under ROC.

**Supplemental Table 13. SEN for Cross-validation of HER2-overexpressed & Non-HER2-overexpressed type.**

| **Models** | **Fold 1** | **Fold 2** | **Fold 3** | **Fold 4** | **Fold 5** | **Mean** | **SD** |
| --- | --- | --- | --- | --- | --- | --- | --- |
| **LR** | 0.4167 | 0.4167 | 0.5000 | 0.5000 | 0.6667 | 0.5000 | 0.0913 |
| **RF** | 0.3333 | 0.4167 | 0.4167 | 0.4167 | 0.2500 | 0.3667 | 0.0667 |
| **NB** | 0.2500 | 0.3333 | 0.41667 | 0.5000 | 0.4167 | 0.3833 | 0.0850 |
| **SVM** | 0.2500 | 0.3333 | 0.41667 | 0.5000 | 0.3333 | 0.3667 | 0.0850 |
| **XGBoost** | 0.3333 | 0.5833 | 0.5000 | 0.5833 | 0.5833 | 0.5167 | 0.0972 |

**Note.**—LR=Logistic Regression, RF=Random Forest, NB= Naïve Bayes, SVM=Support Vector Machine, XGBoost=eXtreme Gradient Boosting, SEN=sensitivity.

**Supplemental Table 14. SPE for Cross-validation of HER2-overexpressed & Non-HER2-overexpressed type.**

| **Models** | **Fold 1** | **Fold 2** | **Fold 3** | **Fold 4** | **Fold 5** | **Mean** | **SD** |
| --- | --- | --- | --- | --- | --- | --- | --- |
| **LR** | 0.8148 | 0.7778 | 0.7407 | 0.8889 | 0.7407 | 0.7926 | 0.0620 |
| **RF** | 1.0000 | 0.9630 | 1.0000 | 0.9630 | 0.9259 | 0.9704 | 0.0310 |
| **NB** | 0.8519 | 0.8519 | 0.8519 | 0.8519 | 0.7407 | 0.8296 | 0.0497 |
| **SVM** | 0.9259 | 0.8889 | 0.9260 | 0.9259 | 0.8148 | 0.8963 | 0.0483 |
| **XGBoost** | 0.9630 | 0.9630 | 0.8519 | 0.8519 | 0.9630 | 0.9185 | 0.0609 |

**Note.**—LR=Logistic Regression, RF=Random Forest, NB= Naïve Bayes, SVM=Support Vector Machine, XGBoost=eXtreme Gradient Boosting, SPE=specificity.

**Supplemental Table 15. F1-Score for Cross-validation of HER2-overexpressed & Non-HER2-overexpressed type.**

| **Models** | **Fold 1** | **Fold 2** | **Fold 3** | **Fold 4** | **Fold 5** | **Mean** | **SD** |
| --- | --- | --- | --- | --- | --- | --- | --- |
| **LR** | 0.4545 | 0.4348 | 0.4800 | 0.5714 | 0.5926 | 0.5067 | 0.0710 |
| **RF** | 0.5000 | 0.5556 | 0.5882 | 0.5556 | 0.3529 | 0.5105 | 0.0934 |
| **NB** | 0.3158 | 0.4000 | 0.4762 | 0.5455 | 0.4167 | 0.4308 | 0.0860 |
| **SVM** | 0.3529 | 0.4211 | 0.5263 | 0.6000 | 0.3810 | 0.4563 | 0.1039 |
| **XGBoost** | 0.4706 | 0.7000 | 0.5455 | 0.6087 | 0.7000 | 0.6049 | 0.0996 |

**Note.**—LR=Logistic Regression, RF=Random Forest, NB= Naïve Bayes, SVM=Support Vector Machine, XGBoost=eXtreme Gradient Boosting.

**Supplemental Table 16. PRE for Cross-validation of HER2-overexpressed & Non-HER2-overexpressed type.**

| **Models** | **Fold 1** | **Fold 2** | **Fold 3** | **Fold 4** | **Fold 5** | **Mean** | **SD** |
| --- | --- | --- | --- | --- | --- | --- | --- |
| **LR** | 0.5000 | 0.4545 | 0.4615 | 0.6667 | 0.5333 | 0.5232 | 0.0862 |
| **RF** | 1.0000 | 0.9543 | 1.0000 | 0.9000 | 0.8700 | 0.9449 | 0.0525 |
| **NB** | 0.5678 | 0.65 | 0.5556 | 0.7889 | 0.4167 | 0.5958 | 0.1222 |
| **SVM** | 0.7896 | 0.8907 | 0.8143 | 0.75 | 0.8897 | 0.8269 | 0.0556 |
| **XGBoost** | 0.8000 | 0.8750 | 0.7995 | 0.6365 | 0.8750 | 0.7972 | 0.0871 |

**Note.**—LR=Logistic Regression, RF=Random Forest, NB= Naïve Bayes, SVM=Support Vector Machine, XGBoost=eXtreme Gradient Boosting, PRE=precision.

**Supplemental Table 17. FPR for Cross-validation of HER2-overexpressed & Non-HER2-overexpressed type.**

| **Models** | **Fold 1** | **Fold 2** | **Fold 3** | **Fold 4** | **Fold 5** | **Mean** | **SD** |
| --- | --- | --- | --- | --- | --- | --- | --- |
| **LR** | 0.1852 | 0.2222 | 0.2593 | 0.1111 | 0.2593 | 0.2074 | 0.0554 |
| **RF** | 0 | 0.0370 | 0 | 0.0370 | 0.0741 | 0.0296 | 0.0277 |
| **NB** | 0.1481 | 0.1481 | 0.1481 | 0.1481 | 0.2594 | 0.1704 | 0.0442 |
| **SVM** | 0.0741 | 0.1111 | 0.0741 | 0.0741 | 0.1852 | 0.1037 | 0.0432 |
| **XGBoost** | 0.0370 | 0.0370 | 0.1481 | 0.1481 | 0.037 | 0.0815 | 0.0544 |

**Note.**—LR=Logistic Regression, RF=Random Forest, NB= Naïve Bayes, SVM=Support Vector Machine, XGBoost=eXtreme Gradient Boosting, FPR=false positive rate.

**Supplemental Table 18. GM for Cross-validation of HER2-overexpressed & Non-HER2-overexpressed type.**

| **Models** | **Fold 1** | **Fold 2** | **Fold 3** | **Fold 4** | **Fold 5** | **Mean** | **SD** |
| --- | --- | --- | --- | --- | --- | --- | --- |
| **LR** | 0.5827 | 0.5693 | 0.6086 | 0.4445 | 0.6667 | 0.5744 | 0.0730 |
| **RF** | 0.5773 | 0.6335 | 0.6455 | 0.4013 | 0.6335 | 0.5782 | 0.0916 |
| **NB** | 0.4615 | 0.5329 | 0.5958 | 0.4260 | 0.6526 | 0.5338 | 0.0834 |
| **SVM** | 0.4811 | 0.5443 | 0.6212 | 0.4630 | 0.6804 | 0.5580 | 0.0826 |
| **XGBoost** | 0.5665 | 0.7495 | 0.6526 | 0.4969 | 0.7049 | 0.6341 | 0.0917 |

**Note.**—LR=Logistic Regression, RF=Random Forest, NB= Naïve Bayes, SVM=Support Vector Machine, XGBoost=eXtreme Gradient Boosting, GM=geometric mean.

**Supplemental Table 19. ACC for Cross-validation of HER2-overexpressed & Non-HER2-overexpressed type.**

| **Models** | **Fold 1** | **Fold 2** | **Fold 3** | **Fold 4** | **Fold 5** | **Mean** | **SD** |
| --- | --- | --- | --- | --- | --- | --- | --- |
| **LR** | 0.6923 | 0.6667 | 0.6667 | 0.7692 | 0.7179 | 0.7026 | 0.0429 |
| **RF** | 0.7948 | 0.7949 | 0.8205 | 0.7949 | 0.7179 | 0.7862 | 0.0389 |
| **NB** | 0.6667 | 0.6923 | 0.7180 | 0.7436 | 0.6410 | 0.6923 | 0.0405 |
| **SVM** | 0.7179 | 0.7180 | 0.7692 | 0.7949 | 0.6667 | 0.7333 | 0.0500 |
| **XGBoost** | 0.7692 | 0.8462 | 0.7436 | 0.769 | 0.8462 | 0.7949 | 0.0480 |

**Note.**—LR=Logistic Regression, RF=Random Forest, NB= Naïve Bayes, SVM=Support Vector Machine, XGBoost=eXtreme Gradient Boosting, ACC=accuracy.

**Supplemental Table 20. AUC for Cross-validation of TN & Non-TN type.**

| **Models** | **Fold 1** | **Fold 2** | **Fold 3** | **Fold 4** | **Fold 5** | **Mean** | **SD** |
| --- | --- | --- | --- | --- | --- | --- | --- |
| **LR** | 0.8202 | 0.7709 | 0.7877 | 0.7223 | 0.7854 | 0.7773 | 0.0356 |
| **RF** | 0.9005 | 0.8590 | 0.8698 | 0.8314 | 0.9003 | 0.8722 | 0.0293 |
| **NB** | 0.7854 | 0.7595 | 0.7270 | 0.6843 | 0.7693 | 0.7451 | 0.0401 |
| **SVM** | 0.8915 | 0.8508 | 0.8837 | 0.8645 | 0.8809 | 0.8743 | 0.0164 |
| **XGBoost** | 0.9203 | 0.9185 | 0.9350 | 0.9152 | 0.9410 | 0.9260 | 0.0113 |

**Note.**—LR=Logistic Regression, RF=Random Forest, NB= Naïve Bayes, SVM=Support Vector Machine, XGBoost=eXtreme Gradient Boosting, AUC= area under ROC, TN=Triple-negative.

**Supplemental Table 21. ACC for Cross-validation of TN & Non-TN type.**

| **Models** | **Fold 1** | **Fold 2** | **Fold 3** | **Fold 4** | **Fold 5** | **Mean** | **SD** |
| --- | --- | --- | --- | --- | --- | --- | --- |
| **LR** | 0.8507 | 0.8358 | 0.8433 | 0.8134 | 0.8358 | 0.8358 | 0.0140 |
| **RF** | 0.8507 | 0.8507 | 0.8433 | 0.8433 | 0.8582 | 0.8493 | 0.0062 |
| **NB** | 0.7985 | 0.8060 | 0.7985 | 0.7836 | 0.8284 | 0.8030 | 0.0163 |
| **SVM** | 0.8582 | 0.8507 | 0.8507 | 0.8507 | 0.8657 | 0.8552 | 0.0067 |
| **XGBoost** | 0.8881 | 0.8657 | 0.8731 | 0.8731 | 0.8731 | 0.8746 | 0.0082 |

**Note.**—LR=Logistic Regression, RF=Random Forest, NB= Naïve Bayes, SVM=Support Vector Machine, XGBoost=eXtreme Gradient Boosting, ACC=accuracy, TN=Triple-negative.

**Supplemental Table 22. SEN for Cross-validation of TN & Non-TN type.**

| **Models** | **Fold 1** | **Fold 2** | **Fold 3** | **Fold 4** | **Fold 5** | **Mean** | **SD** |
| --- | --- | --- | --- | --- | --- | --- | --- |
| **LR** | 0.3913 | 0.2609 | 0.3478 | 0.2174 | 0.3043 | 0.3043 | 0.0615 |
| **RF** | 0.1304 | 0.1304 | 0.0870 | 0.0870 | 0.1740 | 0.1218 | 0.0325 |
| **NB** | 0.3913 | 0.2609 | 0.2609 | 0.3043 | 0.3913 | 0.3217 | 0.0590 |
| **SVM** | 0.1739 | 0.1304 | 0.1304 | 0.1304 | 0.2174 | 0.1565 | 0.0348 |
| **XGBoost** | 0.4783 | 0.3478 | 0.3913 | 0.3478 | 0.4348 | 0.4000 | 0.0507 |

**Note.**—LR=Logistic Regression, RF=Random Forest, NB= Naïve Bayes, SVM=Support Vector Machine, XGBoost=eXtreme Gradient Boosting, SEN=sensitivity,TN=Triple-negative.

**Supplemental Table 23. SPE for Cross-validation of TN & Non-TN type.**

| **Models** | **Fold 1** | **Fold 2** | **Fold 3** | **Fold 4** | **Fold 5** | **Mean** | **SD** |
| --- | --- | --- | --- | --- | --- | --- | --- |
| **LR** | 0.9460 | 0.9550 | 0.9459 | 0.9369 | 0.9459 | 0.9459 | 0.0064 |
| **RF** | 1.0000 | 1.0000 | 1.0000 | 1.0000 | 1.0000 | 1.0000 | 0 |
| **NB** | 0.8829 | 0.9189 | 0.9099 | 0.8829 | 0.9189 | 0.9027 | 0.0185 |
| **SVM** | 1.0000 | 1.0000 | 1.0000 | 1.0000 | 1.0000 | 1.0000 | 0 |
| **XGBoost** | 0.9730 | 0.9730 | 0.9730 | 0.9820 | 0.9640 | 0.9730 | 0.0064 |

**Note.**—LR=Logistic Regression, RF=Random Forest, NB= Naïve Bayes, SVM=Support Vector Machine, XGBoost=eXtreme Gradient Boosting, SPE=specificity, TN=Triple-negative.

**Supplemental Table 24. PRE for Cross-validation of TN & Non-TN type.**

| **Models** | **Fold 1** | **Fold 2** | **Fold 3** | **Fold 4** | **Fold 5** | **Mean** | **SD** |
| --- | --- | --- | --- | --- | --- | --- | --- |
| **LR** | 0.6000 | 0.5454 | 0.5714 | 0.4167 | 0.5385 | 0.5344 | 0.0701 |
| **RF** | 1.0000 | 1.0000 | 1.0000 | 1.0000 | 1.0000 | 1.0000 | 0 |
| **NB** | 0.4091 | 0.4000 | 0.3750 | 0.3500 | 0.5000 | 0.4068 | 0.0570 |
| **SVM** | 1.0000 | 1.0000 | 1.0000 | 1.0000 | 1.0000 | 1.0000 | 0 |
| **XGBoost** | 0.7857 | 0.7273 | 0.7500 | 0.8000 | 0.7143 | 0.7555 | 0.03681 |

**Note.**—LR=Logistic Regression, RF=Random Forest, NB= Naïve Bayes, SVM=Support Vector Machine, XGBoost=eXtreme Gradient Boosting, PRE=precision, TN=Triple-negative.

**Supplemental Table 25. F1-Score for Cross-validation of TN & Non-TN type.**

| **Models** | **Fold 1** | **Fold 2** | **Fold 3** | **Fold 4** | **Fold 5** | **Mean** | **SD** |
| --- | --- | --- | --- | --- | --- | --- | --- |
| **LR** | 0.4737 | 0.3529 | 0.4324 | 0.2857 | 0.3889 | 0.3867 | 0.0725 |
| **RF** | 0.2308 | 0.2308 | 0.1600 | 0.1600 | 0.2963 | 0.2156 | 0.0573 |
| **NB** | 0.4000 | 0.3158 | 0.3077 | 0.3256 | 0.4390 | 0.3576 | 0.0585 |
| **SVM** | 0.2963 | 0.2308 | 0.2308 | 0.2308 | 0.3571 | 0.2691 | 0.0568 |
| **XGBoost** | 0.5946 | 0.4706 | 0.5143 | 0.4848 | 0.5405 | 0.5210 | 0.0492 |

**Note.**—LR=Logistic Regression, RF=Random Forest, NB= Naïve Bayes, SVM=Support Vector Machine, XGBoost=eXtreme Gradient Boosting,TN=Triple-negative.

**Supplemental Table 26. FPR for Cross-validation of TN & Non-TN type.**

| **Models** | **Fold 1** | **Fold 2** | **Fold 3** | **Fold 4** | **Fold 5** | **Mean** | **SD** |
| --- | --- | --- | --- | --- | --- | --- | --- |
| **LR** | 0.0541 | 0.0450 | 0.0541 | 0.0631 | 0.0541 | 0.0541 | 0.0052 |
| **RF** | 0 | 0 | 0 | 0 | 0 | 0 | 0 |
| **NB** | 0.1171 | 0.0811 | 0.0901 | 0.1171 | 0.0811 | 0.0973 | 0.0151 |
| **SVM** | 0 | 0 | 0 | 0 | 0 | 0 | 0 |
| **XGBoost** | 0.0270 | 0.0270 | 0.0270 | 0.0180 | 0.0360 | 0.0270 | 0.0052 |

**Note.**—LR=Logistic Regression, RF=Random Forest, NB= Naïve Bayes, SVM=Support Vector Machine, XGBoost=eXtreme Gradient Boosting, FPR=false positive rate, TN=Triple-negative..

**Supplemental Table 27. GM for Cross-validation of TN & Non-TN type.**

| **Models** | **Fold 1** | **Fold 2** | **Fold 3** | **Fold 4** | **Fold 5** | **Mean** | **SD** |
| --- | --- | --- | --- | --- | --- | --- | --- |
| **LR** | 0.6084 | 0.4992 | 0.7065 | 0.8405 | 0.9168 | 0.7143 | 0.1513 |
| **RF** | 0.3611 | 0.3611 | 0.6009 | 0.7752 | 0.8805 | 0.5958 | 0.2114 |
| **NB** | 0.5878 | 0.4896 | 0.6997 | 0.8365 | 0.9146 | 0.7057 | 0.1558 |
| **SVM** | 0.4170 | 0.3611 | 0.6009 | 0.7752 | 0.8805 | 0.6069 | 0.1998 |
| **XGBoost** | 0.6822 | 0.5817 | 0.7627 | 0.8733 | 0.9345 | 0.7669 | 0.1272 |

**Note.**—LR=Logistic Regression, RF=Random Forest, NB= Naïve Bayes, SVM=Support Vector Machine, XGBoost=eXtreme Gradient Boosting, GM=geometric mean, TN=Triple-negative.
